# Development and Testing of a Field Diagnostic Assay for Peste des Petits Ruminants Virus

**DOI:** 10.1111/tbed.12266

**Published:** 2014-07-30

**Authors:** J Baron, E Fishbourne, E Couacy-Hyman, M Abubakar, B A Jones, L Frost, R Herbert, T R Chibssa, G van't Klooster, M Afzal, C Ayebazibwe, P Toye, J Bashiruddin, M D Baron

**Affiliations:** 1 The Pirbright InstitutePirbright, UK; 2 Virology laboratoryBingerville, Cote-d'Ivoire (Ivory Coast); 3 National Veterinary LaboratoryIslamabad, Pakistan; 4 Royal Veterinary CollegeHatfield, UK; 5 National Animal Health Diagnostic and Investigation Center (NAHDIC)Sebeta, Ethiopia; 6 FAO EthiopiaAddis Abeba, Ethiopia; 7 FAO OfficeIslamabad, Pakistan; 8 National Animal Disease Diagnostics and Epidemiology Centre (NADDEC), Ministry of Agriculture, Animal Industry and FisheriesEntebbe, Uganda; 9 International Livestock Research InstituteNairobi, Kenya

**Keywords:** emerging diseases, diagnostics, disease control, virus

## Abstract

We have developed an immunochromatographic test for the diagnosis of peste des petits ruminants (PPR) under field conditions. The diagnostic assay has been tested in the laboratory and also under field conditions in Ivory Coast, Pakistan, Ethiopia and Uganda. The test is carried out on a superficial swab sample (ocular or nasal) and showed a sensitivity of 84% relative to PCR. The specificity was 95% over all nasal and ocular samples. The test detected as little as 10^3^ TCID_50_ (50% tissue culture infectious doses) of cell culture-grown virus, and detected virus isolates representing all four known genetic lineages of peste des petits ruminants virus. Virus could be detected in swabs from animals as early as 4 days post-infection, at a time when clinical signs were minimal. Feedback from field trials was uniformly positive, suggesting that this diagnostic tool may be useful for current efforts to control the spread of PPR.

## Introduction

Peste des petits ruminants (PPR) is a viral disease of sheep and goats which has shown rapid spread throughout large parts of the developing world in the last 20 years (Banyard et al., 2010; Kwiatek et al., 2011). It is now found in most countries in Africa as far south as Tanzania, throughout the Near and Middle East and in large parts of Asia, including a recent outbreak in China that has reached from the western border with Tajikistan to the eastern borders with Russia and North Korea in <4 months (OIE, 2014). The disease is characterized by fever, congestion in, and discharge from, the eyes and nose, respiratory problems, erosive sores in the mouth, lesions around the outside of the mouth, possibly swelling of the lips and diarrhoea, although not all signs are seen in all infected animals. Mortality can be up to 80%, although it is more commonly around 30%, and morbidity is very high (>90%) (reviewed in Baron et al., 2011; Albina et al., 2013; Couacy-Hymann et al., 2007). The economic impact of the disease, and its propensity for rapid spread through the movement of infected animals, has led to the disease being notifiable to the World Organisation for Animal Health (OIE).

One of the problems limiting efforts to control the spread of the disease is that of quickly and correctly identifying outbreaks. The disease is often unfamiliar to livestock keepers in communities dependent on sheep or goats. The set of clinical signs elicited by PPR virus (PPRV) can vary depending on the breed of host and the strain of virus, and it can be hard to distinguish PPR from other diseases of small ruminants such as ‘orf’, contagious caprine pleuropneumonia (CCPP) or bluetongue. This means that veterinarians, to take disease-appropriate action, must either be able to correctly identify the disease from the signs, based on experience, or wait for samples to be sent to a regional or national laboratory and for the results of laboratory tests to be sent back, which may take several days. This kind of delay will inevitably lead to increased dispersion of the disease, as firm control measures will not be applied until a definitive diagnosis is available. In addition, the separation in time and distance of field sampling and final laboratory test result decreases the incentive for field workers to submit samples, leading to inefficient monitoring and delayed diagnosis.

This situation would be alleviated to some extent if diagnosis could be made in the field/at the pen side. For PPRV, the currently available diagnostic tests (agarose gel immunodiffusion (AGID), enzyme-linked immunosorbent assay (ELISA), reverse transcription–polymerase chain reaction (RT-PCR), either gel-based or real-time, or loop-mediated isothermal amplification (LAMP) (OIE, 2012)) vary in their technical demands, but all require a laboratory, particularly for molecular techniques such as RT-PCR or LAMP. We have developed a pen side test for PPRV based on immunochromatographic lateral flow technology (reviewed in Posthuma-Trumpie et al., 2009). The device has been tested in the laboratory, under controlled conditions with experimentally infected animals and in the field during actual or suspected outbreaks in several countries where PPR is endemic. The test has proven itself reliable and sensitive and will provide an important tool in the campaign to control and eventually roll back the spread of this disease.

## Materials and Methods

Isolates of PPRV used in laboratory tests and tests with experimental animals were from the archive of viruses available at The Pirbright Institute and were grown and titred in Vero cells expressing the canine form of the morbillivirus receptor, Signalling Lymphocyte Activation Molecule (SLAM) (CD150), as previously described (Chinnakannan et al., 2013). Confirmatory PCR assays were carried out using published procedures (Forsyth and Barrett, 1995; Couacy-Hymann et al., 2002; Batten et al., 2011), although different tests were used in different laboratories.

All animal studies were carried out under project licences issued by the UK Home Office, after approval by The Pirbright Institute Animal Welfare and Ethical Review Body (AWERB). All staff carrying out procedures on animals had appropriate personal licences issued by the UK Home Office.

The lateral flow device (LFD)-based test for PPRV was manufactured by Foresite Diagnostics Ltd, Sand Hutton, York, UK, using monoclonal antibody C77 recognizing the H protein of PPRV (Anderson et al., 1990; Anderson and McKay, 1994). The stocks of C77 were prepared using hybridoma cells grown in a miniPerm bioreactor and purified on a Protein G HiTrap column (GE Healthcare Life Sciences, Bucks, UK).

Swab samples from animals were taken on artificial fibre (‘Floq’) or cotton swabs; material from the swabs was eluted in 500–600 ul of LFD buffer ‘TBCE’ (Foresite) and 80–100 ul of the eluate applied to the test strip. Preparations of cell culture-grown viruses were diluted as required into LFD buffer such that 80 ul contained the required dose of virus, which was applied to the test. Tissue extracts were prepared from samples stored at −80°C; approx. 0.5 g tissue was ground in a mortar with 30 drops of LFD buffer, the material transferred to a test tube along with a further 20 drops LFD buffer which was used to wash the mortar, and spun at 2500 ***g*** for 5 mins. Six drops of the supernatant were transferred to a LFD and developed as normal.

Developed tests were scanned or photographed after 15–30 mins. Scans/photographs were rotated and cropped as required in Adobe Photoshop.

## Results and Discussion

### Prototype development and laboratory testing

**Figure 1:**
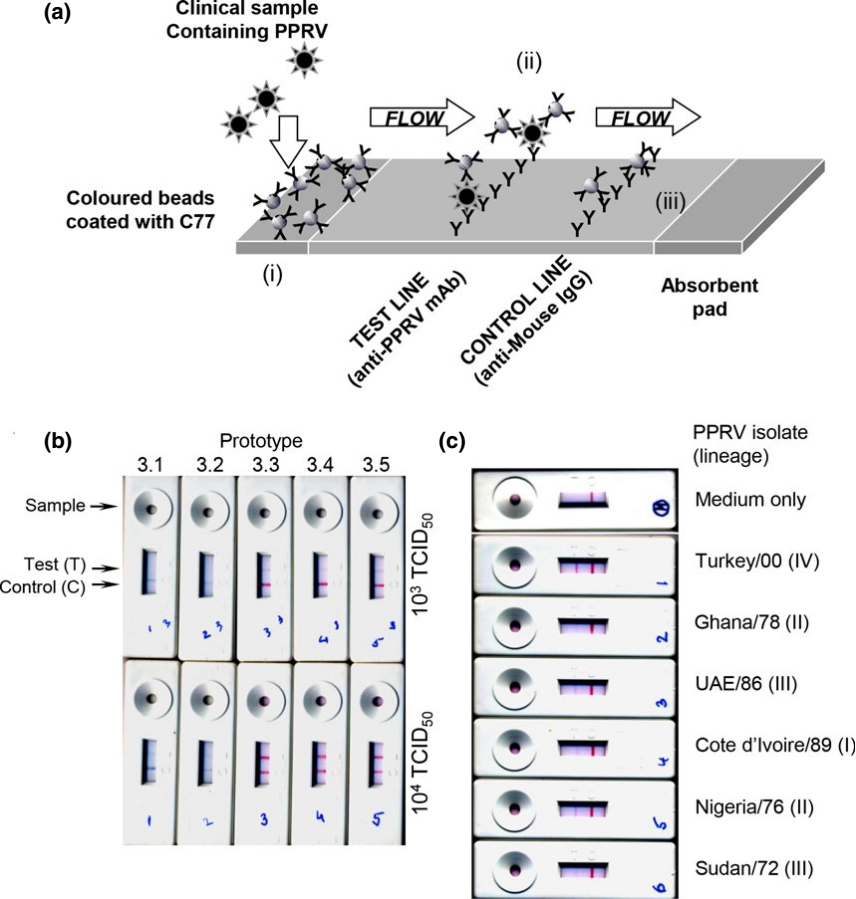
Screening and laboratory testing of lateral flow device (LFD)-based assay. (a) Basic operation of the immunochromatographic assay. Sample is added to the test port where it mixes with beads in the reagent pad (i). Any virus antigen in the sample binds to beads there. Beads are carried by chromatographic flow along the test strip to the test line, where they encounter further anti-PPRV antibody (ii). Any virus or virus antigen bound to the beads will be immobilized on the test line, thereby immobilizing some of the beads and creating a positive signal. Remaining beads are carried further along the strip until they come to the control line, where they are bound by anti-mouse IgG antibody. (b) Screening of prototypes 3.1–3.5 with 10^3^ and 10^4^ TCID_50_ of PPRV (strain Sudan/72). (c) Confirmation of the ability of the device to recognize virus from all known lineages. Prototype 3.5 was used to screen 10^3^ TCID_50_ of cell culture-grown virus of each strain.

The LFD-based PPRV test is based on the specificity and affinity of monoclonal antibody C77. This antibody recognizes the PPRV H protein, but not that of the related ruminant morbillivirus rinderpest (Anderson et al., 1990; Anderson and McKay, 1994; Das et al., 2000); it is the basis of a widely used competition ELISA for PPRV antibodies (Anderson et al., 1990; Anderson and McKay, 1994) and has previously been used in a prototype penside test for PPRV (Brüning-Richardson et al., 2011). This monoclonal antibody is used both as the trapping reagent on the chromatographic test strip and to decorate the detection beads (Fig.1a), which are either latex (blue) or colloidal gold (red) (Fig.1b). The specificity of the antibody thereby ensures the specificity of the test.

In collaboration with the manufacturers, we screened a large number of prototype tests to see which combination of filter strip, buffer and beads would give a sufficiently sensitive test. In determining the criteria by which we would assess prototype tests, we estimated the amount of virus that might be found in swabs from PPRV-infected animals. Previous studies in the reference laboratory at The Pirbright Institute had shown that our real-time PCR assay (Batten et al., 2011) gave a similar result with extracts from an ocular or nasal swab as seen with 10^3^ TCID_50_ of cell culture-grown PPRV. While such a swab might contain more viral antigen than found in this amount of cell culture-grown PPRV, we reasoned it was unlikely to contain less, as swab material would contain also dead cells and other host animal material which could contain non-infectious or no longer infectious viral antigen. We therefore set detection of 10^3^ TCID_50_ of PPRV as our primary criterion for acceptability of a test. Two prototypes from the third batch of tests (3.3 and 3.5) showed clear positive results with this amount of PPRV (Fig.1b). These prototypes recognized PPRV isolates representing all four known lineages of the virus (Fig.1c), as expected from the known ability of C77 to recognize all lineages of PPRV. The variation observed in the intensity of the ‘Test’ band obtained with different isolates was found to depend on the dilution of virus stock required to give 10^3^ TCID_50_ in 80 ul; the greater the dilution, the weaker the band seen in the test. This observation is in accord with our experience that laboratory-grown stocks of virus contain variable amounts of non-infectious viral antigen and RNA as well as protein and RNA derived from the cultured cells. Normal preparations of tissue culture-grown virus contain significant amounts of microparticulate cell debris from the freeze–thaw cycling of the cells, which is used to release the virus; it is probable that this material contains viral antigen which can contribute to the positive result in the test. It cannot be excluded that some strain-specific variation in sensitivity of the test exists, which may occur within or between lineages. This may be the result of antigenic variation or of differences between strains in the excretion of virus antigen into eye or nose discharge. The latter possibility is a problem that affects all kinds of tests detecting virus antigen. While such variations in sensitivity with strain may occur, it does not appear to be sufficient to prevent the test detecting virus from a range of different countries and of different genetic lineages.

**Figure 2:**
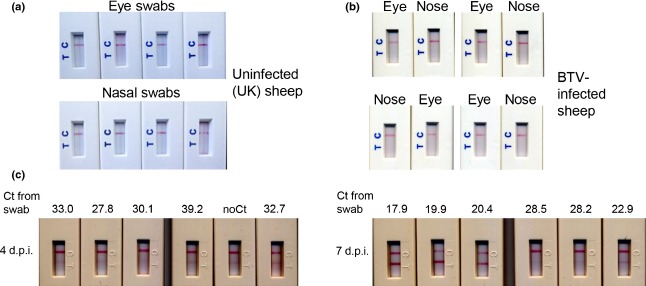
Testing of lateral flow device (LFD) based on animal samples (a) Examples of eye swabs or nasal swabs from uninfected UK sheep tested on the LFD-based assays. (b) Examples of eye swabs or nasal swabs taken from animals with severe bluetongue and tested on the LFD-based assays. (c) Examples of nasal swabs taken from UK goats infected with PPRV. Swabs were taken 4 or 7 dpi as indicated and tested on the LFD-based assays; the remaining eluate from each swab was tested for PPRV genome by real-time PCR, and the result recorded as the *C*_t_ determined in the PCR assay.

The LFD-based test was applied to swabs taken from a number of UK sheep and goats as negative controls, as there is no known PPR in the UK, nor has there ever been. All these tests gave negative results, as expected (Fig.2a). (Note that different batches of the test came mounted in slightly different plastic cases; this did not affect the internal contents of the tests nor their performance). In addition, no positive result was seen when the test was used on swabs from UK sheep that had been experimentally infected with bluetongue virus (BTV) (Fig.2b); BTV infection can give rise to similar signs as PPRV (fever, nasal discharge) and could easily give rise to suspicion of PPRV in the field. We took the swabs at the stage of strong clinical signs, but no positive result was observed in the PPRV test.

Lateral flow device-based tests were also used on samples from animals experimentally infected with PPRV (Baron et al., 2014). At 4 days post-infection (dpi), most animals were showing a rise in rectal temperatures, but no other clinical signs, so they would be unlikely to have been spotted as infected by a livestock keeper in field conditions. At 7 dpi, the animals were showing nasal and ocular congestion, and some animals had a slight ocular or nasal discharge. Positive results from the LFD were seen in some animals at 4 dpi, and in all animals at 7 dpi (Fig.2c). These observations showed that the test can detect PPRV even at early stages of infection. The remaining swab eluate from these samples was assayed for PPRV genome RNA by real-time PCR; the swab eluates were also found to be positive for virus by PCR, although perfect correlation was not observed between the swab results and the PCR test, especially at 4 dpi. This may have been due to the LFD buffer not being optimized for RNA preservation, or variation in the ratio of viral membrane antigen to viral genome in different animals.

### Field testing of the PPRV penside test

Peste des petits ruminants virus penside tests were distributed to a number of centres in countries where PPRV is, or has recently been, prevalent (Pakistan, Ethiopia, Ivory Coast, Uganda). Tests were used to assay fresh or archived swabs, or in one case to assay extracts of frozen tissue from experimental animals. Swabs were taken primarily from eye or nose, although some samples were taken from mouth or faeces. In all cases, the result from the test was confirmed using gel-based or real-time PCR, depending on the laboratory. These results, together with results from animals tested in the UK, are collected in Table1. As can be seen, there was very good correlation between the test result and the result from the corresponding PCR of the same sample. In a small number of cases, usually associated with a weak PCR result, the pen side test failed to detect PPRV, but this was expected, as the penside test is essentially a pocket form of immunocapture ELISA, and was not going to be as sensitive as PCR, which can detect a few 10 s of copies of the virus genome. The calculated sensitivity, relative to PCR, and taken over the ocular and nasal swabs, was 84%, with a calculated specificity of 95%. We did observe a small fraction of nasal swabs, although no eye swabs, which were unambiguously positive for PPRV in the penside test, but negative in PCR. It is unknown whether this is due to genuine false positives from nasal samples, or some component of nasal mucous which is inhibitory in RT-PCR in the same way that heparin is known to be (Beutler et al., 1990; Holodniy et al., 1991).

**Table 1:** Compilation of results of lateral flow device (LFD) (penside) PPRV test compared with laboratory test results. For each test used, we tabulated the type of sample and the result (+/−) of the LFD test and the corresponding PCR test for PPRV. Assuming that the PCR test is a definitive test for infection with PPRV, the sensitivity of the test for ocular/nasal swabs was 83.54% (95% CI: 73.5–90.9%) while the specificity was 94.59% (95% CI: 86.7–98.5%)

Sample type	Sample origin	Number of samples	PCR neg; LFD neg	PCR neg; LFD pos	PCR pos; LFD neg	PCR pos; LFD pos
Nasal swabs	UK (known clean)^a^	12	12	0	–	–
	UK (known −ve)^a^	10	10	0	–	–
	UK (known +ve)^a^	27	0	1	4	22
	Ivory Coast^b^	18	9	0	0	9
	Uganda^b^	8	1	2	0	5
	Pakistan^c^	21	4	1	3	13
Eye swabs	UK(known clean)^a^	8	8	0	–	–
	UK (known −ve)^a^	5	5	0	–	–
	Ivory Coast^b^	16	7	0	1	8
	Ethiopia^b^	10	10	0	0	0
	Pakistan^c^	18	4	0	5	9
Oral swabs	Ivory Coast^b^	10	2	0	4	4
Faecal swabs	Pakistan^c^	10	5	0	3	2
Tissue homogenate	Ethiopia^b^	9	0	0	2	7

a PCR tests for PPRV were carried out in the UK using real-time PCR as described in Batten et al. (2011). Note that, for UK animals that were known to be free of PPRV, the PCR test was not carried out but can be assumed to be negative.

b PCR tests for PPRV were carried out in Ivory Coast, Ethiopia and Uganda using conventional (gel-based) PCR as described in Couacy-Hymann et al. (2002).

c PCR tests for PPRV were carried out in Pakistan using real-time PCR as described in Kwiatek et al. (2010).

For a number of animals, replicate samples were taken from eye and nose, and in some cases from mouth or faeces. These data are summarized in Table2. No significant difference was seen in the sensitivity of nasal and oral swabs, although both were superior to oral or faecal swabs. In general, these observations suggest eye swabs are the most useful sample type to be used in the field, especially if the sample is to be transferred to a laboratory for further testing (e.g. PCR for sequencing and lineage determination).

**Table 2:** Detailed results from animals sampled at multiple points. Animals 1–8 were from Pakistan; animals 9–17 were from Ivory Coast. PCR status (whether the animal was infected with PPRV or not according to PCR-based tests on nasal and eye swabs) was determined using the assays given in Table1

Animal	Status (PCR)	LFD			
		Nasal	Eye	Oral	Faecal
1	+ve	+ve	+ve	ND	−ve
2	+ve	+ve	−ve	ND	−ve
3	+ve	+ve	−ve	ND	+ve
4	+ve	−ve	+ve	ND	ND
5	+ve	+ve	+ve	ND	−ve
6	+ve	−ve	−ve	ND	−ve
7	+ve	+ve	+ve	ND	ND
8	+ve	+ve	+ve	−ve	ND
9	+ve	+ve	+ve	−ve	ND
10	+ve	+ve	+ve	+ve	ND
11	+ve	+ve	+ve	+ve	ND
12	+ve	+ve	+ve	−ve	ND
13	−ve	−ve	−ve	−ve	ND
14	−ve	−ve	−ve	−ve	ND
15	+ve	+ve	+ve	−ve	ND
16	+ve	+ve	+ve	+ve	ND
17	+ve	+ve	+ve	+ve	ND

No problem was reported in carrying out the test away from the laboratory. No extra equipment is required apart from the swabs, a tube containing LFD buffer and a plastic transfer pipette for applying the swab extract to the test. One group subdivided the kit, which contains 25 tests, to provide packets of 2–5 tests to be used on any one field visit.

The test was also able to identify PPRV in extracts of frozen tissues (Table1), although this reduces the test to the status of laboratory assay, where it offers no advantage over the traditional icELISA, and is more expensive. Using the test in this way may, in some cases, be convenient, for example if it is necessary to use in an emergency in laboratories where normal assays for PPRV have not yet been established.

An acknowledged criticism of this sort of test is that the result can be subjective; while negatives have no line at the T position, and strong positives are very clear, it is possible for samples with little virus (e.g. some of the samples at 4 dpi in Fig.2) to be open to question as to whether they are positive or not. Objective evaluation of the test results would require an optical or electrochemical reader or other device. This would probably raise the sensitivity of the test, but the need for such a device would greatly raise the cost of the test as well as make it impractical for field use in developing countries. The benefits of using the penside test are primarily the ability to confirm or deny PPRV infection far more quickly than would be possible using laboratory-based testing. This means that the implementation of movement controls and the initiation of vaccination or stamping out can be put in place with the minimum delay. Where clinical knowledge and experience of PPR is limited, for example in countries or areas where the disease has only recently arrived, or where outbreaks have been too sporadic to build up local knowledge, a negative result can be important in removing concerns of a new PPR outbreak. The test gives a clear result, which could be easily photographed using a smart phone, such as are common even in developing world countries, and the photograph transmitted back to a central veterinary office or laboratory as confirmation that a reported disease is or is not PPR. The test does not require special training and could be used by livestock keepers, veterinary technicians (paravets) or veterinary officers, depending on local need. It is expected that this test will prove a useful addition to the tools available, given the extensive spread of PPR over the past 10 years, and growing interest in establishing proper control of this economically very important disease (Baron et al., 2011; Albina et al., 2013).
